# Reduced Whole‐Brain Network Segregation and Dorsal Anterior‐Cingulate Cortex Neurochemical Alterations in Chronic Smokers

**DOI:** 10.1002/brb3.71391

**Published:** 2026-04-24

**Authors:** Humberto Monsivais, Francesco Versace, Max Wintermark, Brian A. Taylor

**Affiliations:** ^1^ Department of Imaging Physics The University of Texas MD Anderson Cancer Center Houston Texas USA; ^2^ Department of Behavioral Science The University of Texas MD Anderson Cancer Center Houston Texas USA; ^3^ Department of Neuroradiology The University of Texas at MD Anderson Cancer Center Houston Texas USA

**Keywords:** anterior cingulate cortex, graph theory, proton magnetic resonance spectroscopy, smokers, structural covariance network, structural MRI

## Abstract

**Background:**

Chronic tobacco use has been linked to alterations in brain structure, yet how these changes are organized across large‐scale networks and relate to underlying neurochemistry remains unclear. This study combined structural magnetic resonance imaging (MRI) with proton magnetic resonance spectroscopy (^1^H‐MRS) to examine network organization and neurochemical alterations in smokers.

**Methods:**

Fifty‐one smokers and 51 non‐smokers underwent whole‐brain structural MRI and single‐voxel ^1^H‐MRS in the dorsal anterior cingulate cortex (dACC), a hub within executive‐control and salience networks. Individualized structural covariance networks were constructed from cortical thickness values, and graph theory was applied to evaluate global and regional properties.

**Results:**

At the network level, smokers exhibited reduced modularity across multiple densities, indicating weaker segregation of brain systems with otherwise preserved global topology. Exploratory cortical thickness analyses suggested diffuse thinning in smokers, although these effects did not survive correction for multiple comparisons. At the neurochemical level, smokers demonstrated lower total N‐acetylaspartate (tNAA) in the dACC (Cliff's δ = 0.35), consistent with reduced neuronal integrity. No group differences were observed for glutamine, glutathione, or GABA. Regional analyses revealed reduced centrality in executive‐control areas and relatively increased centrality in sensory and reward‐related regions. Exploratory correlations between smoking severity, network metrics, and tNAA were weak and did not survive multiple comparisons correction.

**Conclusion:**

Chronic smoking was associated with reduced network segregation and lower dACC tNAA values. By incorporating whole‐brain network analyses with regionally specific spectroscopy in our study, we add a multiscale perspective on vulnerability in smokers and reinforce the importance of longitudinal, regionally specific studies.

## Graphical abstract

Chronic smokers exhibited less segregated brain networks and shifts in centrality from executive to reward‐related regions. Additional spectroscopy measures revealed lower tNAA in the dorsal anterior cingulate cortex, highlighting network‐level and neurochemical alterations that may underlie smoking‐related vulnerability.

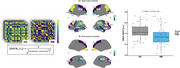

## 1 Introduction

Tobacco use is a leading cause of premature mortality worldwide and significantly elevates risk for cancer, respiratory, and cardiovascular disease (World Health Organization 2023; Branstetter et al. 2016; Fagerström 2002; Shen et al. 2018). Yet despite widespread public health campaigns and the availability of treatments, quitting smoking remains difficult, and many individuals relapse over time. These challenges are thought to reflect lasting changes in brain circuits that regulate reward, self‐control, and emotional processing, underscoring the importance of characterizing neural mechanisms underlying tobacco dependence for developing more effective and targeted interventions to aid smoking cessation.

Neuroimaging studies have documented smoking‐related changes using both functional magnetic resonance imaging (fMRI) and structural MRI (Beltz et al. 2015; Vergara et al. 2017; Weidler et al. 2024; Weng et al. 2021). While functional connectivity offers insights into real‐time brain dynamics, structural imaging provides complementary information by capturing more stable, long‐term changes in brain morphology (Alexander‐Bloch et al. 2013; Evans 2013; Khundrakpam et al. 2013). Structural studies consistently report reduced gray matter volume and cortical thinning in smokers across multiple regions, including the prefrontal cortex, anterior cingulate cortex (ACC), insula, thalamus, cerebellum, and temporal lobes (Khundrakpam et al. 2013; Chen et al. 2021; Durazzo et al. 2018; Kühn et al. 2010; Li et al. 2015; Peng et al. 2018), which may contribute to impaired executive function, impulsivity, and altered emotional regulation.

Proton magnetic resonance spectroscopy (^1^H‐MRS) studies complement structural imaging by quantifying neurochemical markers such as N‐acetylaspartate (NAA), glutamate, glutamine, and gamma‐aminobutyric acid (GABA) within key regions like the hippocampus, amygdala, nucleus accumbens, dorsal ACC, and prefrontal cortex (Durazzo et al. 2015; Steinegger et al. 2024; Steinegger et al. 2021; O'Neill et al. 2023; Gutzeit et al. 2013; Mennecke et al. 2014; Schulte et al. 2017). Metabolites like NAA and choline‐containing compounds may change before measurable structural decline (Meyerhoff et al. 2013), thus serving as markers of neuronal integrity and excitatory/inhibitory balance (D'Souza and Markou 2013; Goldstein et al. 2009; Kenny and Markou 2004). Given the key role of the ACC in cognitive control and reward‐related processing, its structural and neurochemical integrity is of particular interest in addiction disorders.

Traditional morphometric approaches, including region‐of‐interest and voxel‐based morphometry, are limited in capturing inter‐regional relationships. Structural covariance networks (SCNs) address this by assessing coordinated morphology across the entire brain rather than isolating single regions (Gong et al. 2012). SCNs can be derived from cortical surface area and thickness, which together contribute to gray matter volume but can independently reveal structural abnormalities even when overall volume appears unaffected (Ducharme et al. 2016; Winkler et al. 2018). SCNs have become a useful tool for examining the anatomical organization of large‐scale brain systems, particularly in the context of neurological and psychiatric conditions, including obsessive‐compulsive disorder (Yun et al. 2016), Parkinson's disease (Diao et al. 2024; Zhou et al. 2020), Alzheimer's disease (Montembeault et al. 2016; Qing et al. 2021), and schizophrenia (Prasad et al. 2022; Wannan et al. 2019). When combined with graph theory, SCNs enable quantification of both global network architecture (e.g., modularity, clustering, small‐worldness) and regional influence (e.g., centrality measures), providing a systems‐level view of structural brain networks (Bullmore and Sporns 2012; Sporns 2013). Although smokers have been studied with functional connectivity and diffusion imaging (Tan et al. 2019; Zhang et al. 2018), graph‐theoretical analysis of cortical thickness‐based SCNs has not yet been reported.

SCNs provide a systems‐level perspective on how cortical regions are organized, while ^1^H‐MRS allows a closer look at the local neurochemical environment that may contribute to (or reflect) these large‐scale alterations. We focused on the dACC for spectroscopy because it serves as a hub within executive control and salience networks, repeatedly implicated in smoking, including cue reactivity, craving regulation, nicotine dependence, and withdrawal‐related alterations in functional connectivity (Abulseoud et al. 2020; Lerman et al. 2014; Zhao et al. 2012). As such, the dACC provides a biologically motivated region in which to examine smoking‐related neurochemical differences.

Thus, while structural and neurochemical abnormalities in smokers have been documented, the relationship between large‐scale brain network organization and local neurochemistry remains unclear. In this study, we applied an individualized SCN approach to examine graph‐theoretical properties of cortical thickness networks in smokers. We then tested neurochemical alterations in the dorsal ACC using ^1^H‐MRS, a region central to cognitive control and reward processing. Together, these analyses offer a multiscale characterization of how chronic smoking disrupts brain structure and neurochemistry.

## 2 Methods and Materials

### 2.1 Subjects Recruitment

This study was approved by the Institutional Review Board (IRB) of The University of Texas at MD Anderson Cancer Center (IRB 2020‐0824), and informed consent was obtained from all participants before study sessions. A total of 51 healthy controls and 51 smokers were recruited from the Houston area through social media and printed advertisements. Inclusion criteria for all participants included males and females (smokers and non‐smokers) between 21 and 65 years of age, and be able to speak and read English. Participants were classified as smokers if they met the following criteria. First, during the prescreening interview, they had to report having smoked ≥100 cigarettes in their lifetime and smoking at least five cigarettes per day. Although the ≥100‐cigarette threshold is not empirically derived, it is a widely used epidemiological criterion (Bondy et al. 2009) that when combined with the five‐cigarettes‐per‐day requirement, helped ensure that the sample included regular smokers rather than occasional users or individuals who had only experimented with cigarettes. Then, on the day of the MRI scan, participants had to self‐report daily cigarette smoking, and recent nicotine exposure was biochemically verified using a urinary cotinine test, a widely used biomarker of tobacco use (Benowitz et al. 2009). Together, these criteria ensured that individuals classified as smokers represented active, established cigarette smokers at the time of participation. For non‐smokers, participants must not have smoked more than 100 cigarettes in their lifetime, test negative for cotinine using a urine test, attest to being a non‐smoker (self‐report) on the day of MR imaging, and report not using electronic cigarettes or other tobacco products in the last 30 days. The demographics of all participants are presented in Table 1.

**TABLE 1:** Demographic characteristics.

	Non‐smokers (*n* = 51)	Smokers (*n* = 51)	*p* value^a^
Mean age (SD)	40 (13)	48 (12)	0.30
Females	45%	51%	
Race			
Asian	16%	0%	
Black or African American	27%	49%	
Native Hawaiian/Pacific Islander	0%	2%	
White	51%	39%	
More than one race	6%	8%	
Prefer not to say	0%	2%	
Questionnaire scores			
			
FTND	—	4.2 (2.3)	
Years of smoking	—	29.6 (13.5)	
QSU	—	46.3 (26.5)	
BIS_attention_	12.8 (2.4)	13.0 (3.5)	0.42
BIS_motor_	20.0 (2.6)	21.4 (4.5)	**0.03**
BIS_nonplanning_	20.8 (4.6)	22.7 (5.3)	**0.03**
PANAS_negative_	14.1 (4.3)	16.0 (7.1)	0.06
PANAS_positive_	35.9 (6.9)	35.0 (7.2)	0.27
SHAPS	4.9 (4.1)	5.4 (5.0)	0.24

Abbreviation: BIS, Barratt impulsiveness scale; FTND, Fagerström test for nicotine dependence; PANAS, positive and negative affect scale; QSU, questionnaire for smoking urges; SHAPS, Snaith–Hamilton pleasure scale.

^a^Student *t*‐test.

Participants were excluded if they had MRI contraindications (e.g., pacemakers, neurostimulators), current diagnosis or treatment for major psychiatric disorders (e.g., depression, bipolar disorder, schizophrenia, PTSD), high alcohol use (>14 drinks/week), substance abuse treatment, positive pregnancy test, significant sensory impairments, recent seizures or epilepsy, or a concussion with loss of consciousness within the past 6 months.

### 2.2 Self‐Report Measures

On the day of the MRI visit, all participants completed a battery of validated self‐report measures. The Fagerström Test for Nicotine Dependence [FTND, (Heatherton et al. 1991)] assessed nicotine dependence severity on a scale of 0–10. The Questionnaire for Smoking Urges [QSU‐Brief, (Cox et al. 2001)] is a 10‐item scale measuring smoking cravings. The Barratt Impulsiveness Scale [BIS‐11, (Patton et al. 1995)] is a 30‐item measure of trait impulsivity across cognitive, motor, and non‐planning domains. The Positive and Negative Affect Schedule [PANAS, (Watson and Clark 1997)] includes 20 items measuring current emotional states, yielding separate positive and negative affect scores. The Snaith–Hamilton Pleasure Scale [SHAPS, (Snaith et al. 1995)] is a 14‐item instrument measuring hedonic capacity, where higher scores indicate greater impairment in the ability to experience pleasure.

### 2.3 MRI and MRS Data Acquisition

All participants underwent brain scans on a whole‐body 3T Prisma MRI System (Siemens Healthineers, Erlangen, Germany) using a vendor‐supplied 32‐channel receiver head coil. T1‐weighted high‐resolution structural images were acquired using a 3D MPRAGE sequence (1×1×1.2 mm resolution) for anatomical reference, placement of the MRS voxel, and subsequent tissue fraction extraction from the voxel. MRS acquisitions included a PRESS (Bottomley 1987) sequence; TR/TE = 2000/80, 128 averages, TA = 4.91 min, for NAA and glutamate detection, and HERMES (Chan et al. 2016; Chan et al. 2017; Saleh et al. 2016; Saleh et al. 2019) sequence; TR/TE = 2000/80, 320 averages, “ON”/“OFF” editing pulse frequency = 1.9/7.5 ppm, TA = 10.7 min, for GABA and glutathione (GSH) detection from a 25 × 40 × 27 mm^3^ voxel placed in the dorsal anterior cingulate cortex (dACC). Water reference scans (8 averages for PRESS, 16 for HERMES) with the same acquisition parameters but without water suppression were acquired for eddy current correction and water scaling.

### 2.4 ^1^H‐MRS Analysis

^1^H‐MRS raw data were pre‐processed using Osprey (Oeltzschner et al. 2020) [v2.7.0] and Gannet (Edden et al. 2014) [v3.3.2] toolboxes for PRESS and HERMES, respectively. Both toolboxes processed data per expert consensus recommendations (Near et al. 2020), including coil‐combination, frequency and phase correction, averaging, eddy current correction, and residual water removal. Specifically, HERMES spectra were zero‐filled to 32768 data points, and a 3‐Hz exponential line broadening filter was applied before fitting and quantification. PRESS data were fitted and quantified using the LCModel (Provencher 2001) wrapper built into Osprey. Final quantification results are reported as water‐scaled values corrected for cerebralspinal fluid (CSF).

### 2.5 Intra‐Individual Cortical Covariance Networks

**FIGURE 1:**
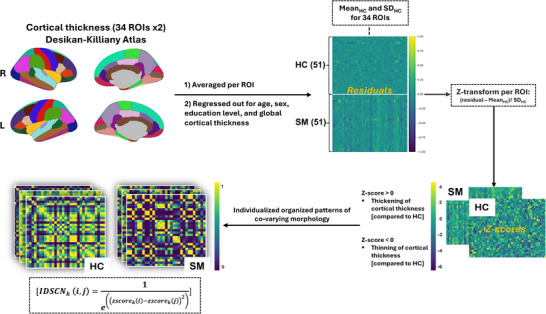
Overview of study procedures: construction of intra‐individual brain structural covariance networks for graph analysis. $IDSCN_{k}\left(i,j\right)$ refers to the individualized structural covariance network (joint variation) between the ith (i = 1–34) and the jth (j = 1–34) ROI for the kth (k = 1–125) participant, $z-score_{k}\left(i\right)$ is the *z*‐transformed value of the ith ROI of cGMT for the kth participant, and $z-score_{k}\left(j\right)$ is the *z*‐transformed value of the jth ROI of cortical grey matter thickness for the kth participant. Adapted from Yun et al. 2020. HC, healthy controls; L, left; R, right; ROI, region of interest; SD, standard deviation; SM, smokers.

Intra‐individual structural covariance networks (IDSCNs) were constructed at the individual level using the method described in Yun et al. 2020. Briefly, mean cortical gray matter thicknesses (cGMTs) were calculated from 34 cortical regions of interest per hemisphere defined by the Desikan–Killiany atlas (Desikan et al. 2006). The mean values were corrected for age, sex, education level, and mean global cortical thickness. Although total intracranial volume (TIV) is correlated with surface area of the cortex (Winkler et al. 2010), TIV is not correlated with the average cortical thickness; thus, we chose to use the mean cortical thickness of the global brain area as recommended by Goto et al. 2022. The residuals were standardized using *z*‐score transformation, with the mean and standard deviation derived from the healthy control group for each ROI. This process quantified the extent of morphological variation in brain regions relative to the average values observed in healthy individuals. Next, a measure of joint variation was used to determine the edge weights of the networks. These weights, ranging between 0 and 1, were computed based on the 34 cGMT values using the method described by Yun et al. 2016; Yun et al. 2020. In summary, the IDSCNs essentially represent a “similarity map” where edge weights reflect the similarity between two brain regions’ change in cortical thickness based on their z‐score deviation from healthy controls (Wee et al. 2013). An overview of the construction of intra‐individual brain structural covariance networks is presented in Figure 1.

### 2.6 Global Network Characteristics, Hub Profiling, and Regional Network Characteristics

Graph network analysis was carried out using the Brain Connectivity Toolbox (Rubinov and Sporns 2010) in MATLAB R2024a. IDSCNs underwent thresholding and binarization across network density values from *K* = 0.05 to 0.30, increasing in increments of 0.1. This approach helped eliminate spurious connections by preserving only the most robust edges in the matrix. Global network properties were examined through metrics of network segregation (clustering coefficients and modularity), network integration (global efficiency), and their equilibrium (small‐worldness). Across the network density levels (*K* = 0.05–0.30), the criteria outlined by Uehara et al. 2014 were met predominantly within the narrower range of *K* = 0.18–0.25. Specifically, more than 50% of the networks satisfied three key conditions: (i) network connectedness, where over 80% of nodes remained linked within the network; (ii) modular organization, with a modularity score exceeding 0.3; and (iii) small‐world characteristics, indicated by a small‐worldness value greater than 1. Thus, the average of the narrower range (*K* = 0.22) was selected for group comparison of global and regional network characteristics. Principal brain regions serving as key indicators of morphological alterations were identified through hub profiling using rank‐transformed betweenness, eigenvector, and closeness centrality measures. An exploratory surface‐based morphometry analysis is presented in Figure S1 and Table 1.

### 2.7 Statistical Analysis

MRS values were compared across the healthy control (HC) and smoker (SM) groups using the Wilcoxon rank‐sum test (since not all values were normally distributed) in RStudio (Posit team 2025), with significant findings corrected via Benjamini–Hochberg false discovery rate (FDR) correction (*α* = 0.05). Outliers were identified via the standard boxplot method. The total NAA (tNAA) values were used in this step, as this is a very well‐defined peak in the spectrum. Since age can play a significant role in tNAA levels, an analysis of covariance (ANCOVA) test was run to compare tNAA values between the groups with age as a covariate. To quantify the magnitude of group differences, we report nonparametric effect sizes using Cliff's delta (δ) (Cliff 1993), a nonparametric effect‐size measure aligned with rank‐based group comparisons, with 95% confidence intervals where appropriate.

Statistical analyses of graph metrics were conducted in MATLAB R2024a. Because network metrics (global and regional) do not follow a normal distribution, group comparisons were done using a Mann–Whitney *U* test with FDR Correction (*α* = 0.05). Global network metrics: global clustering, global efficiency, global modularity, and small‐worldness were compared at the mean of the narrow density range of *K* = 0.22 since it was at this density that over 85% of all participants passed the Uehara criterion described in Section 2.6. For the regional network metrics—betweenness, eigenvector, and closeness centralities—these metrics were also calculated across the narrow density range, averaged, and rank‐transformed using the *tiedrank.m* function in MATLAB. Next, group comparisons were made using a Mann–Whitney *U* test with FDR Correction (*α* = 0.05). The median and interquartile values were also calculated for each group. Detailed statistics are presented in Table 2.

**TABLE 2:** Descriptive statistics of eigenvector and betweenness centrality metrics that show significant differences between the HC and SM groups.

Eigenvector centrality				
Brain region	HC median (IQR)	SM Median (IQR)	*p* value	*p*‐adj (FDR‐corr)
Decreased (SM < HC)				
cACC	0.080 (−0.004–0.164)	0.004 (−0.013–0.021)	0.011	0.037
FUS	0.180 (0.054–0.306)	0.038 (−0.033–0.110)	0.009	0.033
LOC	0.156 (0.030–0.282)	0.024 (−0.032–0.079)	0.019	0.047
periCal	0.203 (0.102–0.304)	0.047 (−0.011–0.104)	<0.001	<0.001
preC	0.180 (0.064–0.296)	0.059 (−0.044–0.163)	<0.001	0.003
rMFG^a^	0.163 (0.053–0.273)	0.004 (−0.064–0.073)	0.024	0.055
SFG	0.063 (−0.049–0.175)	0.001 (−0.013–0.014)	0.003	0.016
STG	0.182 (0.055–0.309)	0.034 (−0.041–0.108)	<0.001	0.003
FP	0.121 (−0.010–0.252)	0.037 (−0.000–0.073)	0.002	0.013
Increased (SM > HC)				
cMFG	0.025 (−0.049–0.100)	0.271 (0.184–0.359)	<0.001	0.003
ENT^a^	0.101 (0.019–0.183)	0.142 (0.000–0.284)	0.03	0.064
MOF	0.031 (−0.035–0.096)	0.138 (0.017–0.259)	0.001	0.007
MTG	0.061 (−0.060–0.182)	0.092 (−0.041–0.224)	0.015	0.042
PCUN	0.107 (−0.007–0.222)	0.249 (0.140–0.359)	0.008	0.033
sMAR	0.036 (−0.044–0.117)	0.206 (0.076–0.336)	0.016	0.042
TP	0.033 (−0.057–0.124)	0.133 (−0.000–0.267)	0.016	0.042
Betweenness centrality				
Decreased (SM < HC)				
rMFG^a^	30.459 (1.452–59.467)	4.335 (−18.421–27.090)	0.025	0.169
SFG	42.383 (−0.717–85.484)	0.001 (−25.964–25.964)	0.005	0.016
sMAR^a^	24.750 (−2.785–52.285)	59.276 (15.292–103.260)	0.006	0.070
Increased (SM > HC)				
MOF	24.544 (−2.338–51.426)	56.912 (0.925–112.898)	0.001	0.021

*Note*: Median, interquartile (IQR) ranges, and *p* values before and after FDR correction are presented.

Abbreviations: cACC, caudal anterior cingulate cortex; cMFG, caudal middle frontal gyrus; ENT, entorhinal; FP, frontal pole; FUS, fusiform gyrus; L, left; LOC, lateral occipital cortex; MOF, medial orbitofrontal gyrus; MTG, middle temporal gyrus; periCal, pericalcarine; PCUN, precuneus cortex; R, right; rMFG, rostral middle frontal gyrus; SFG, superior frontal gyrus; STG, superior temporal gyrus; sMAR, supramarginal gyrus; TP, temporal pole.

^a^ Did not survive multiple comparison correction (FDR at a = 0.05).

To examine associations between imaging measures and behavioral variables, we used nonparametric correlation analyses with Spearman's rank correlation coefficient, as multiple variables were not normally distributed. The correlation was conducted at an individual level, with regional network metrics and tNAA metabolite levels assessed against clinical/behavioral measures (e.g., smoking severity, impulsivity, affective scales). Age, sex, years of education, and mean global cortical thickness were included as covariates, and partial Spearman correlations were calculated accordingly.

## 3 Results

A total of 102 participants (51 smokers, 51 non‐smokers) were recruited. Smokers were slightly older on average than non‐smokers (48 ± 12 vs. 40 ± 13 years), but not statistically different (*p* = 0.30). Age was included as a covariate in all statistical analyses. The groups were matched for sex distribution. As expected, smokers had substantial histories of cigarette use (mean duration: 29.6 ± 13.5 years) and moderate nicotine dependence based on FTND scores (mean = 4.2 ± 2.3). Scores on the QSU, BIS, PANAS, and SHAPS are summarized in Table 1. Smokers demonstrated statistically significantly higher BIS motor and BIS non‐planning scores, and had higher negative affect and impulsivity (PANAS scores), but not significantly, when compared to the controls.

### 3.1 Neurochemical Alterations

**FIGURE 2:**
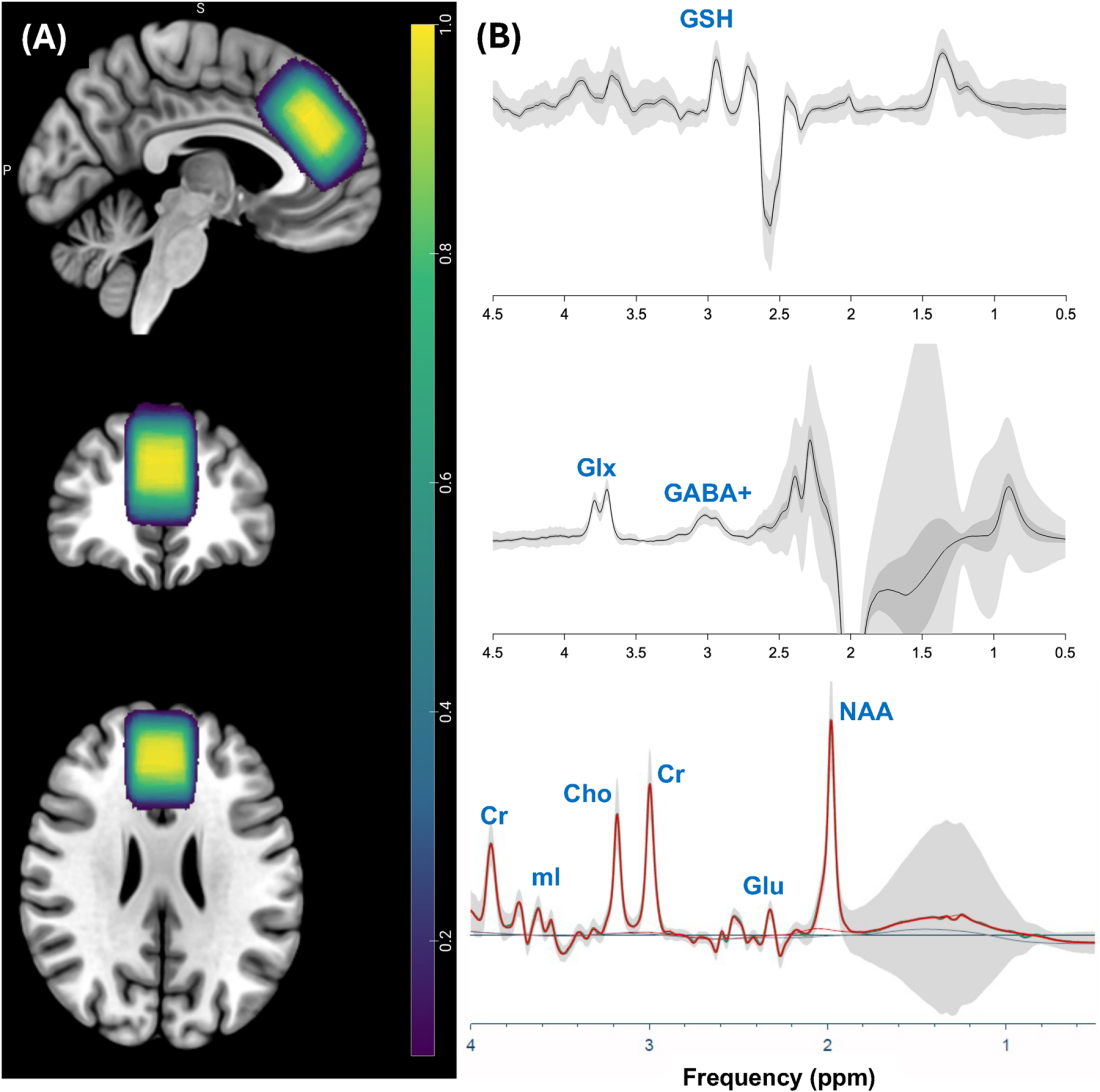
Overview of the MRS data. (A) Consistency map of voxel placement superimposed on the MNI152 template, demonstrates high reproducibility for the dorsal anterior cingulate cortex (dACC). (B) Mean and standard deviation HERMES spectra (top and middle) and PRESS (bottom) from the dACC voxel (TE = 80 ms).

**FIGURE 3:**
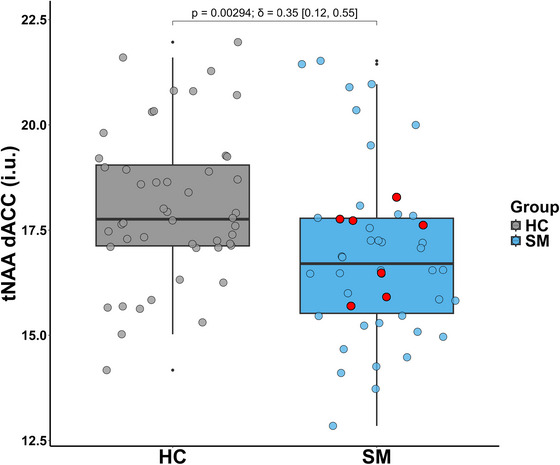
Boxplot shows statistically significant different levels of tNAA between the HC (n = 48) and SM (n = 46) groups. Uncorrected Wilcoxon p‐value shown along with Cliff's δ and its 95% confidence intervals in square brackets. FDR corrected p‐value is 0.01. Filled red circles represent 7 smokers with the highest number of cigarettes smoked per day, as defined in Table S3.

Voxel placement consistency maps and average spectra are presented in Figure 2. After excluding outliers from the statistical analyses (three from the HC group and five from the SM group), the total NAA (tNAA), as measured by PRESS, was significantly decreased in the SM (*n* = 46) group compared to the HC group (*n* = 48). Median NAA+NAAG levels were 16.7 i.u. (IQR = 2.27) in the smoker group and 17.8 i.u. (IQR = 1.92) in the control group. A Wilcoxon rank‐sum test (with W denoting the sum of ranks for one group) indicated a significant difference between groups (*W* = 1494, *p* = 0.0029, *p*‐adj = 0.012, Cliff's δ = 0.35, 95% CI [0.12, 0.55], Figure 3). The ANCOVA test with age as a covariate showed significant differences between the two groups (*p* = 0.014). No statistically significant group differences were found in glutamate, GSH, or GABA (see Figure S4).

### 3.2 Global Network Characteristics

Compared to the HC group, the SM group showed lower modularity at every network density in the narrow density range (*K* = 0.18–0.25, all *p*’s‐adj < 0.05). There were no statistically significant differences in global clustering, efficiency, or small‐worldness.

### 3.3 Regional Network Characteristics

**FIGURE 4:**
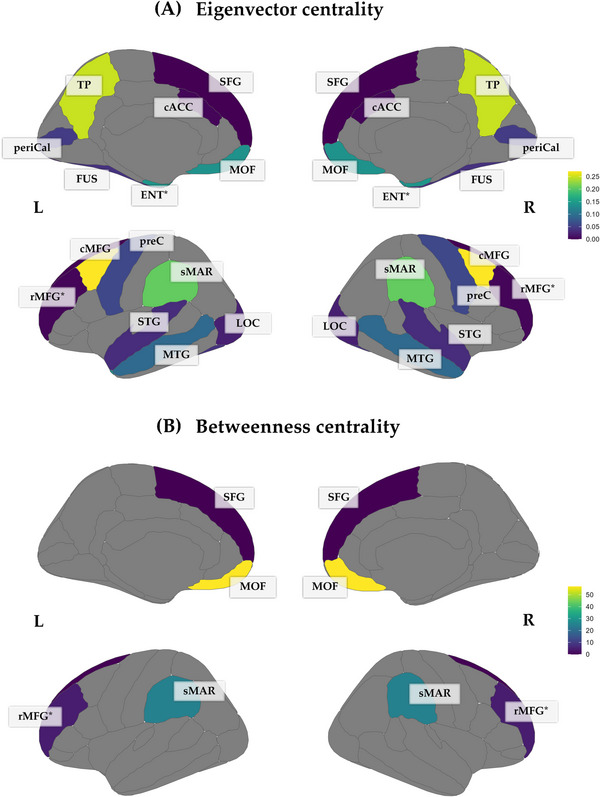
(A) Eigenvector centrality disruption in smokers when compared to controls. The colored regions indicate statistically significant differences between the SM and HC groups, and the color scale represents the median eigenvector centrality value: increased eigenvector centrality (greenish‐yellow colors) and decreased eigenvector centrality (blueish‐purple colors). (B) represents the same as in (A) except for betweenness centrality. Abbreviations: R (right), L (left), caudal anterior cingulate cortex (cACC), fusiform gyrus (FUS), lateral occipital cortex (LOC), pericalcarine (periCal), rostral middle frontal gyrus (rMFG), superior frontal gyrus (SFG), superior temporal gyrus (STG), frontal pole (FP), caudal middle frontal gyrus (cMFG), entorhinal (ENT), medial orbitofrontal gyrus (MOF), middle temporal gyrus (MTG), precuneus cortex (PCUN), supramarginal gyrus (sMAR) and temporal pole (TP). Regions with an asterisk (*) represent regions that show significant differences before FDR‐correction. Note that the FP is not shown since the mapped slices did not intersect this region.

Regional metrics analysis showed decreased eigenvector centrality in the caudal anterior cingulate cortex (cACC), fusiform gyrus (FUS), lateral occipital cortex (LOC), pericalcarine (periCal), pre‐central gyri (preC), rostral middle frontal gyrus (rMFG), superior frontal gyrus (SFG), superior temporal gyrus (STG), and the frontal pole (FP) of the SM group when compared to the HC group. Increased eigenvector centrality was found in the caudal middle frontal gyrus (cMFG), entorhinal (ENT), medial orbitofrontal gyrus (MOF), middle temporal gyrus (MTG), precuneus cortex (PCUN), supramarginal gyrus (sMAR), and temporal pole (TP) of the SM group when compared to the HC group. Descriptive statistics of these findings are presented in Table 2. A graphical representation of these results is presented in Figure 4A.

In terms of betweenness centrality, the SM group showed decreased betweenness centrality in the rMFG, SFG, and sMAR, while increased betweenness centrality in the MOF when compared to the HC group. Descriptive statistics of these findings are presented in Table 2. A graphical representation of these results is presented in Figure 4B. Exploratory results from the surface‐based analysis suggest diffuse cortical thinning in smokers, though these differences did not survive correction.

### 3.4 Behavioral Associations

Partial Spearman correlations controlling for age, sex, education, and mean global cortical thickness showed nominal associations between neurochemical, network, and behavioral measures. tNAA correlated with PANAS negative affect (*ρ* = 0.30, *p* = 0.05), eigenvector centrality in the cACC (*ρ* = 0.37, *p* = 0.02), and eigenvector centrality in the PCUN (*ρ* = –0.32, *p* = 0.04). Network–behavior associations included correlations between MTG centrality and BIS attention (ρ = 0.29, *p* = 0.05), LOG centrality and PANAS negative affect (*ρ* = –0.32, *p* = 0.03), and PCUN centrality and BIS motor impulsivity (*ρ* = –0.36, *p* = 0.01). However, none of these correlations remain significant after correcting for multiple comparisons. Results are summarized in Table 3, and a complete correlation matrix is presented in Figure S3.

**TABLE 3:** Summary of partial Spearman rank correlations.

MRS variable	Imaging variable	Behavioral variable	*ρ*_partial_	*p* value	*p*‐adjusted (FDR)
tNAA		PANAS_negative_	0.30	0.05	0.36
tNAA	eigenvector_cACC		0.37	0.02	0.19
tNAA	eigenvector_PCUN		−0.32	0.04	0.29
	eigenvector_MTG	BIS_attention_	0.29	0.05	0.35
	eigenvector_LOG	PANAS_negative_	−0.32	0.03	0.25
	eigenvector_PCUN	BIS_motor_	−0.36	0.01	0.19

## 4 Discussion

By integrating individualized SCN analysis with graph analysis and measuring ^1^H‐MRS, we identified neurochemical alterations and shared gray matter morphology changes associated with smoking. Two main network findings emerged: lower modularity and reduced centrality in executive regions alongside increased centrality in sensory and reward‐related regions. At the neurochemical level, smokers also showed reduced tNAA in the dACC. Together, these results highlight alterations consistent with impaired cognitive control and heightened reward sensitivity.

### 4.1 Neurochemical and Structural Changes

Reduced tNAA in the dACC aligns with previous reports of smoking‐related neurochemical alterations (Kühn et al. 2010; Li et al. 2015; Peng et al. 2018; Durazzo et al. 2015). Our exploratory surface‐based analysis (see Figure S1 and Table 1) indicated diffuse cortical thinning across frontal, temporal, and cingulate cortices, though these effects did not survive multiple comparison correction. While this pattern is consistent with prior reports of cortical thinning in chronic smokers (Kühn et al. 2010; Li et al. 2015), it should be interpreted cautiously and may reflect subtle structural variation in regions essential for executive regulation and reward processing.

The absence of group differences in glutamate (Glu), GABA, and GSH warrants consideration in light of prior literature. MRS investigations of Glu in smokers have produced inconsistent results, with alterations reported in the dACC (O'Neill et al. 2023) but not consistently across cortical regions, suggesting that glutamatergic dysregulation in smoking may be subtle, state‐dependent, or regionally circumscribed. Steinegger et al. 2021 showed state‐dependent metabolite shifts in the nucleus accumbens linked to craving, and Gutzeit et al. 2013 demonstrated dynamic changes across insular subregions during smoking, withdrawal, and substitution, reinforcing that voxel placement and scanning state are important interpretive considerations. GABA findings in smokers are similarly sparse, and nicotine's comparatively modest effects on inhibitory neurotransmission may not be detectable at the whole‐voxel level in the dACC (Janes et al. 2013). GSH differences between smokers and non‐smokers have also not been consistently demonstrated, likely due to low cortical GSH concentrations relative to MRS sensitivity thresholds and high inter‐individual variability (Fisher et al. 2020). Notably, Guer et al. 2006 found prefrontal NAA normalization after 6 months of smoking cessation, indicating that smoking‐related neurochemical changes may be reversible.

Regarding acquisition parameters, TE = 80 ms offers more favorable spectral separation of Glu from glutamine compared to TE = 30 ms, where overlap in the 2.1 to 2.5 ppm region complicates reliable quantification (Ramadan et al. 2013; Bell et al. 2023). Whether this confers a meaningful advantage for detecting between‐group differences in smoking populations remains to be established. Despite current null findings, multi‐metabolite acquisitions targeting Glu, GABA, and GSH remain worthwhile given well‐grounded mechanistic hypotheses linking nicotine to excitatory/inhibitory imbalance and oxidative stress, particularly in studies with larger samples and greater statistical power (O'Neill et al. 2023). Dose‐response relationships between smoking burden and neurochemical disruption are plausible, but they could not be directly evaluated given the range of smoking severity in the present study. Overall, these findings suggest that voxel placement, state‐dependent effects, reversibility of neurochemical changes, and acquisition parameters are important considerations when interpreting MRS‐related changes in smoking.

### 4.2 Global Network Disruption

The finding of lower modularity, despite otherwise preserved network characteristics (i.e., clustering, global efficiency, and small‐worldness), may suggest reduced segregation of brain systems, with otherwise preserved global topology (Yun et al. 2020). Reduced modularity has been reported in SCN analyses across other substance use disorders, including alcohol (Wang et al. 2024) and cannabis (Xu et al. 2024), where it similarly reflects weakened boundaries between cognitive control and reward networks. In the context of smoking, this may reflect a disrupted balance between cognitive control, reward, and impulse regulation networks that are frequently implicated in addiction (Potvin et al. 2015).

### 4.3 Regional Network Metrics

Our regional graph analyses revealed reduced centrality in the ACC and superior frontal gyrus, regions crucial for cognitive control and goal‐directed behavior (Bush et al. 2000; Ridderinkhof et al. 2004). Lower centrality in the fusiform gyrus (FUS) and lateral occipital cortex (LOC) may reflect altered processing of emotionally salient or drug‐related visual cues. Reduced involvement of the pericalcarine (periCal) and precentral gyri (preC) further suggests disruption of sensorimotor integration. Consistent with this, Lin et al. 2022 reported reduced functional connectivity in sensorimotor and visual networks in smokers, with specific disruptions in the preC and occipital regions. These findings support the interpretation that smoking may compromise visual and sensorimotor pathways involved in cue reactivity and motor responses.

We also detected a decrease in eigenvector centrality in the superior temporal gyrus (STG). STG findings in smoking have been mixed across studies (Peng et al. 2018; Gallinat et al. 2006), with some linked to dependence severity (Chen et al. 2021). The decrease in STG eigenvector centrality may reflect individual variability rather than smoking‐specific effect, given its lack of association with dependence severity or behavioral measures in our sample.

In contrast, several regions showed increased centrality in the smoker group, including the medial orbitofrontal cortex (MOF), precuneus (PCUN), supramarginal gyrus (sMAR), and temporal pole (TP). These areas are associated with reward valuation, self‐referential processing, and affective memory (Goldstein et al. 2009; Goldstein and Volkow 2011; Olson et al. 2007). Increased centrality in reward‐related nodes is consistent with SCN findings in cocaine and opioid/codeine dependence (Wang et al. 2023; Hua et al. 2018; Zheng et al. 2025), where heightened connectivity in orbitofrontal and limbic regions (e.g., pallidum, hippocampus) is interpreted as reflecting elevated salience attribution to drug‐related stimuli. The increased centrality in these nodes may reflect a compensatory shift toward reward‐ and cue‐driven processing, potentially at the expense of executive oversight. Notably, increased centrality in the caudal middle frontal gyrus (cMFG) may also suggest functional compensation for deficits in nearby prefrontal areas associated with self‐regulation.

### 4.4 Integration, Behavioral Associations, and Limitations

Our exploratory cortical thickness findings overlapped with regions showing reduced centrality, particularly within the cingulate and frontal cortices (see Figure S1). In fact, many of the regions identified in the network analysis also showed significantly reduced cortical thickness in smokers compared to controls, even when accounting for age (see Table S2). Notably, the network analysis detected disruption even when conventional morphometry showed only trend‐level differences, indicating that covariance‐based methods may be more sensitive to subtle, distributed alterations than regional thickness or volume measures alone. Despite differences in statistical thresholding, the overlap between cortical thinning and network findings (see Figure S2) suggests that these regions may be vulnerable to smoking‐related structural and topological alterations. At the network level, the overall pattern indicates reduced executive influence and greater prominence of reward‐ and stimulus‐driven regions. The reduction in ACC centrality, combined with lower tNAA in the same region, potentially highlights this region's sensitivity to smoking‐related alterations.

Partial correlation analyses were conducted to directly test associations between neurochemical, network, and behavioral measures. Although none of the effects survived correction for multiple comparisons, several relationships emerged in theoretically consistent directions. For example, lower tNAA in the dACC and reduced LOG centrality were associated with higher negative affect (PANAS_negative_), while altered PCUN and MTG centrality correlated with greater impulsivity (BIS_motor_ and BIS_attention_). These patterns align with models of tobacco use that emphasize disrupted executive–emotional regulation and heightened impulsive tendencies.

Several limitations should be acknowledged. First, the cross‐sectional design limits causal inference; observed differences may reflect consequences of smoking or preexisting vulnerabilities. Second, the smoker group contained a wide range of smoking intensity, with many light to moderate smokers (see Table S3), which may have attenuated group differences. Nevertheless, the effect size for dACC tNAA was moderate (Cliff's δ = 0.35), suggesting neurochemical differences despite group heterogeneity. Third, the dACC MRS voxel was large and likely encompassed functionally heterogeneous subregions, including the dACC, pre‐SMA, and midcingulate, complicating interpretation of metabolite levels. Although this voxel size reflects the signal‐to‐noise demands of single‐voxel spectroscopy, particularly for edited acquisitions, it also increases tissue averaging across subregions. Fourth, the surface‐based cortical thickness analysis showed diffuse thinning, but these findings did not survive correction for multiple comparisons. Finally, brain‐behavior associations were weak and did not survive correction, possibly due to sample heterogeneity, inclusion of lighter smokers, reliance on self‐report measures, and modest effect sizes requiring larger samples.

In conclusion, this multimodal study supports the notion that chronic smokers show reduced segregation among large‐scale brain networks as well as lower tNAA levels in the dACC, and regional centrality shifts, suggesting reduced executive engagement and greater reward/cue involvement. Although comparisons of cortical thickness and brain‐behavior correlations did not survive correction for multiple comparisons, smokers showed altered network topology and lower dACC tNAA. These findings suggest ACC involvement in smoking‐related brain differences, although the weak relationships among measures and the fact that MRS was limited to the dACC make it difficult to draw conclusions about regional specificity. These changes appear subtle yet systematic, consistent with models of executive–reward imbalance in nicotine dependence. Future research should adopt longitudinal designs with a focus on ACC subregions, as well as other brain regions, to establish causality, clarify dose–response relationships, and determine whether smoking‐related brain alterations are reversible with sustained abstinence.

## Author Contributions

**Humberto Monsivais**: formal analysis, methodology, visualization, writing – original draft, writing – review and editing, data curation, investigation. **Francesco Versace**: investigation, resources, writing – original draft, writing – review and editing. **Max A. Wintermark**: investigation, writing – review and editing. **Brian A. Taylor**: Conceptualization, data curation, funding acquisition, investigation, project administration, resources, supervision, writing – review and editing.

## Funding

This work is supported by the US National Institutes of Health through grant K23DA049216 (PI: Taylor) and Cancer Center Support Grant P30CA016672.

## Ethics Statement

This study was reviewed and approved by the Institutional Review Board (IRB) of The University of Texas at MD Anderson Cancer Center (IRB 2020‐0824), and written informed consent was obtained from all participants prior to study participation.

## Conflicts of Interest

The authors declare no conflicts of interest.

## Supporting information



Supplementary Information

## Data Availability

The data that support the findings of this study are available from the corresponding author upon reasonable request.
