# Supplementary material for: Reduced Whole‐Brain Network Segregation and Dorsal Anterior‐Cingulate Cortex Neurochemical Alterations in Chronic Smokers
Source: Brain Behav. 2026 Apr 24;16(4):e71391. doi: 10.1002/brb3.71391 (PMC13109037; doi:10.1002/brb3.71391)
Supplement: Supplementary file 1 — Supplementary Information [file BRB3-16-e71391-s001.docx]

# **Supplementary Materials**

## *Surface-based morphometry*

Surface-based morphometry (SBM) was used to estimate the cortical gray matter (GM) thicknesses of brain regions using the Computational Anatomy Toolbox (CAT12, v12.9) in SPM12 (<https://www.fil.ion.ucl.ac.uk/spm/software/spm12/>), following the recommended defaults from the CAT12 manual (<https://neuro-jena.github.io/cat12-help/>). The process involved: (1) spatial normalization of individual structural images to a common stereotactic space (1), (2) segmentation of the normalized images into gray matter (GM), white matter (WM), and CSF (2), and (3) smoothing of the GM images. Following segmentation, the brain was parcellated into the left (LH) and right (RH) hemispheres. Data from the cortical thickness surfaces of the LH and RH were resampled, combined into a single 32k mesh, and smoothed using a 15 mm full width at half-maximum (FWHM) kernel. Data quality was assessed using the display slices and SBM data homogeneity modules in the CAT12 toolbox. cGMTs estimates were extracted from each hemisphere for 34 regions of interests (ROIs) defined by the Desikan-Killiany atlas (3).

Surface-based statistics were conducted on the smoothed cortical GM thickness images using the general linear model in SPM12. Contrast included healthy controls (HC) > smokers (SM) for each surface measure while accounting for age, sex, education level, and mean global cortical thickness to account for their influence on regional cortical GM thickness. Statistical significance was set at p<0.05, adjusted for multiple comparisons using the Benjamini-Hochberg/false-discovery rate (FDR) correction (α = 0.05). Significant clusters (uncorrected for multiple comparisons) were labeled with the Desikan-Killiany atlas (3).


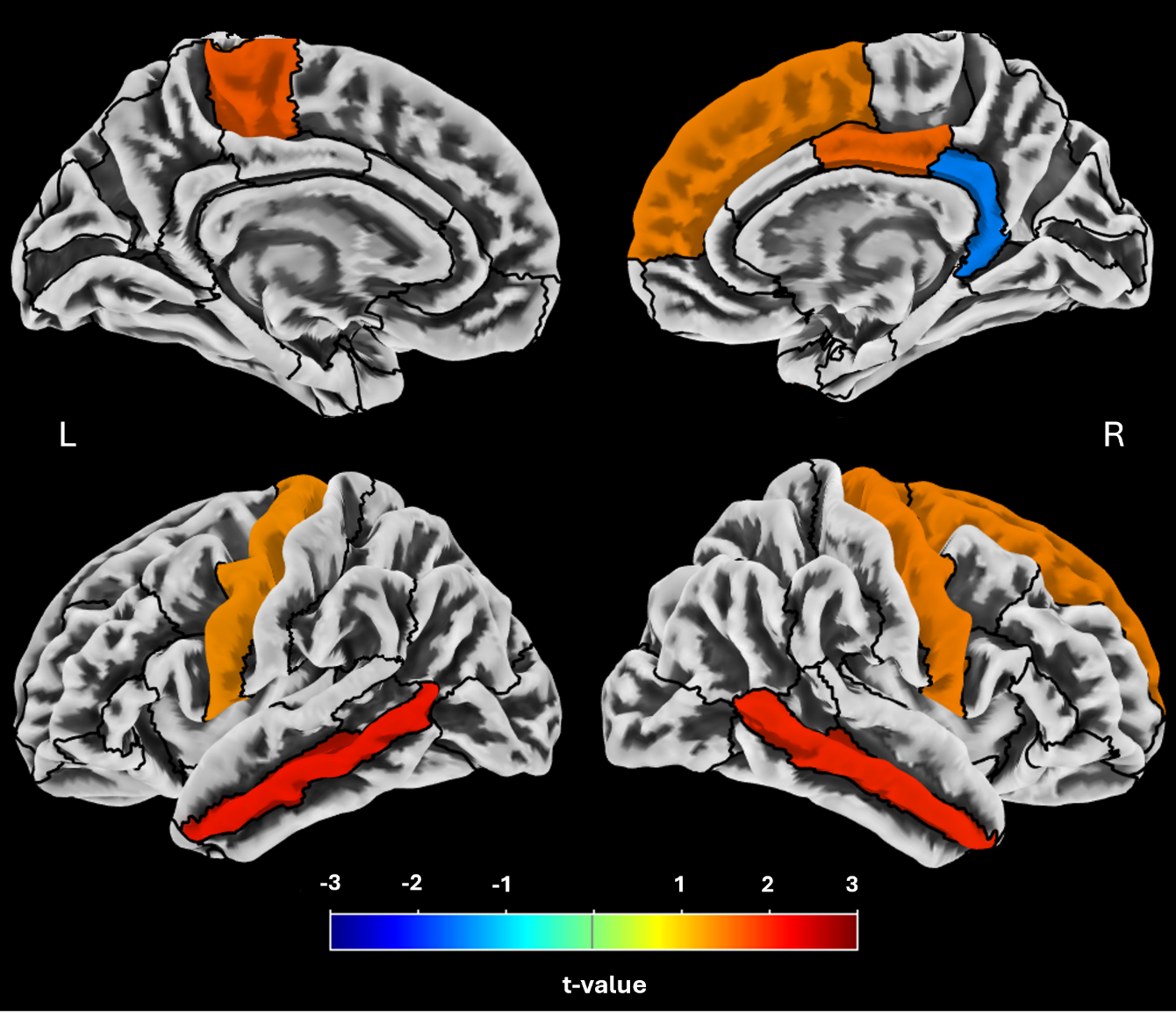


**Supplementary Figure 1**. (A) Regional differences in GM cortical thickness (after controlling for age, sex, education level, and mean global GM cortical thickness). Orange to red colors indicate regions where thinner GM cortical thickness was observed in the smoker group, while blue regions indicate increased GM cortical thickness in the smokers group compared to controls.

**Supplementary Table 1**. Descriptive statistics of SBM results.

| **SM < HC** | **Brain Region** | **Cluster-size**^a^ | **Overlap (%)** | **t-value^b^** | **p-value**^c^ |
| --- | --- | --- | --- | --- | --- |
|  | L middle temporal | 905 | 100 | 2.15 | 0.007 |
|  | L paracentral | 624 | 100 | 1.74 | 0.018 |
|  | L precentral | 654 | 57 | 1.52 | 0.030 |
|  |  |  |  |  |  |
|  | R middle temporal | 994 | 100 | 2.05 | 0.009 |
|  | R superior-frontal  R precentral  R posterior-cingulate | 5003 | 46  41  13 |  | 0.021 |
| **SM > HC** |  |  |  |  |  |
|  | R isthmus cingulate | 497 | 100 | -1.46 | 0.035 |

^a^The number of cortical voxels exhibiting statistically significant differences in thickness between smokers and non-smokers.

^b^ Maximum (peak) t-value within the cluster. The t-values are generated from the voxel-wise regression model.

^c^The cluster size p-value, uncorrected for multiple comparisons, reflects the likelihood of observing a cluster of the given voxel size or larger under the null hypothesis, assuming the brain consists solely of spatially autocorrelated noise.


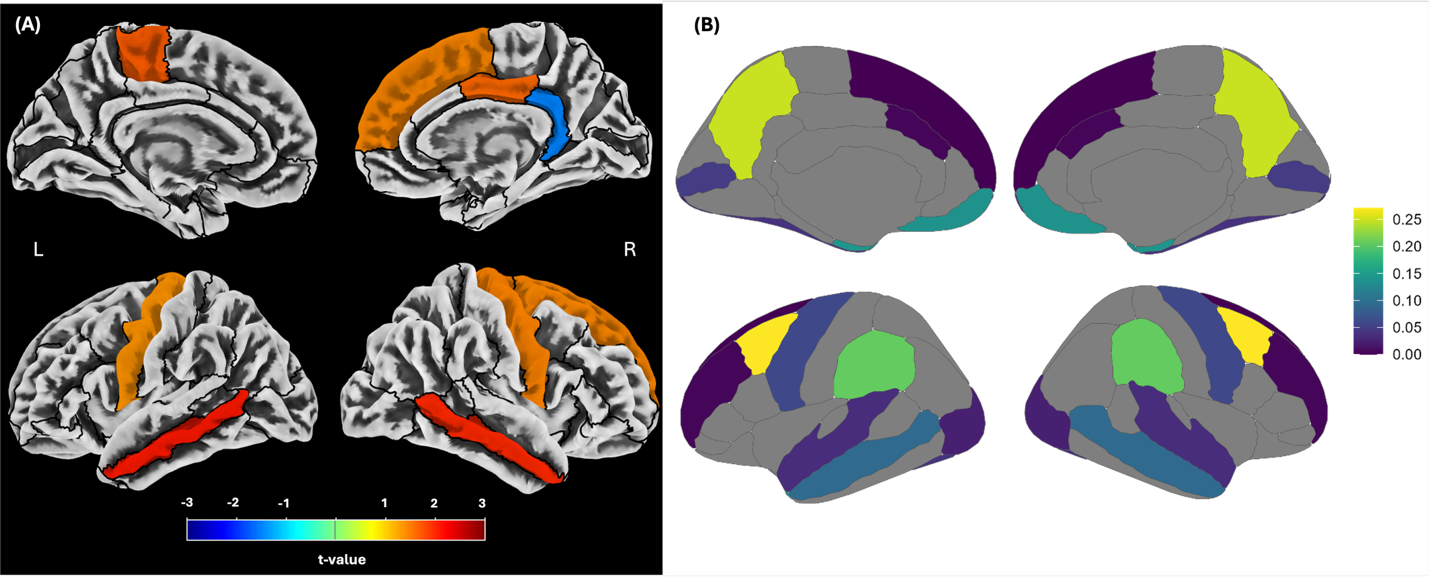


**Supplementary Figure 2**. Overview of altered cortical thickness in smokers (A) and (B) altered network centrality. In (A) orange-red colors indicate thinning of the cortex in smokers while in (B) darker blue-purple indicates decreased eigenvector centrality. Notice the overlap in the superior frontal gyrus (SFG), middle-temporal gyrus (MTG), precentral gyrus (preC), and parts of the anterior cingulate cortex (ACC).

## *Group differences in regional cortical thickness*

To assess group differences in regional cortical thickness while adjusting for covariates, we employed a covariate-adjusted non-parametric approach. Specifically, residuals were obtained from linear regression models in which cortical thickness was regressed on age (a major confounding factor in cortical thickness). Group differences in these residuals were then tested using the Wilcoxon rank-sum test. The selected cortical thickness values come from the findings reported on **Section 3.2** of the manuscript.

**Supplementary Table 2**. Group differences in regional cortical thickness after adjusting for age. Residuals from covariate-adjusted linear models were compared using the Wilcoxon rank-sum test. Reported values include mean and SD, test statistics (W), and p-values.

| Brain Region | HC Mean (SD) | SM Mean (SD) | W | p-value |
| --- | --- | --- | --- | --- |
| periCal | 1.86 (0.12) | 1.76 (0.15) | 1604 | **0.043** |
| cMFG | 2.62 0.13) | 2.53 (0.11) | 1611 | **0.038** |
| preC | 2.48 (0.13) | 2.36 (0.14) | 1764 | **0.002** |
| STG | 2.91 (0.15) | 2.80 (0.14) | 1610 | **0.039** |
| MOF | 2.58 (0.11) | 2.52 (0.12) | 1462 | 0.281 |
| FP | 2.66 (0.15) | 2.61 (0.14) | 1333 | 0.114 |
| SFG | 2.79 (0.13) | 2.70 (0.13) | 1628 | **0.029** |
| FUS | 2.54 (0.10) | 2.49 (0.11) | 1531 | 0.124 |
| PCUN | 2.46 (0.10) | 2.38 (0.12) | 1655 | **0.018** |
| cACC | 2.43 (0.18) | 2.35 (0.18) | 1367 | 0.659 |
| MTG | 2.91 (0.11) | 2.82 (0.10) | 1700 | **0.008** |
| sMAR | 2.56 (0.11) | 2.50 (0.10) | 1462 | 0.281 |
| TP | 3.72 (0.21) | 3.62 (0.24) | 1537 | 0.114 |
| LOC | 2.28 (0.10) | 2.24 (0.08) | 1615 | **0.036** |
| rMFG | 2.62 (0.13) | 2.53 (0.11) | 1340 | 0.794 |
| ENT | 2.50 (0.32) | 2.43 (0.36) | 1353 | 0.728 |

## *Correlation analysis*

Partial Spearman correlation analysis between regional network metrics presented in Section 3.2 and smoking questionaries. Age, sex, years of education, and mean global cortical thickness were included as covariates, and partial Spearman correlations were calculated accordingly.


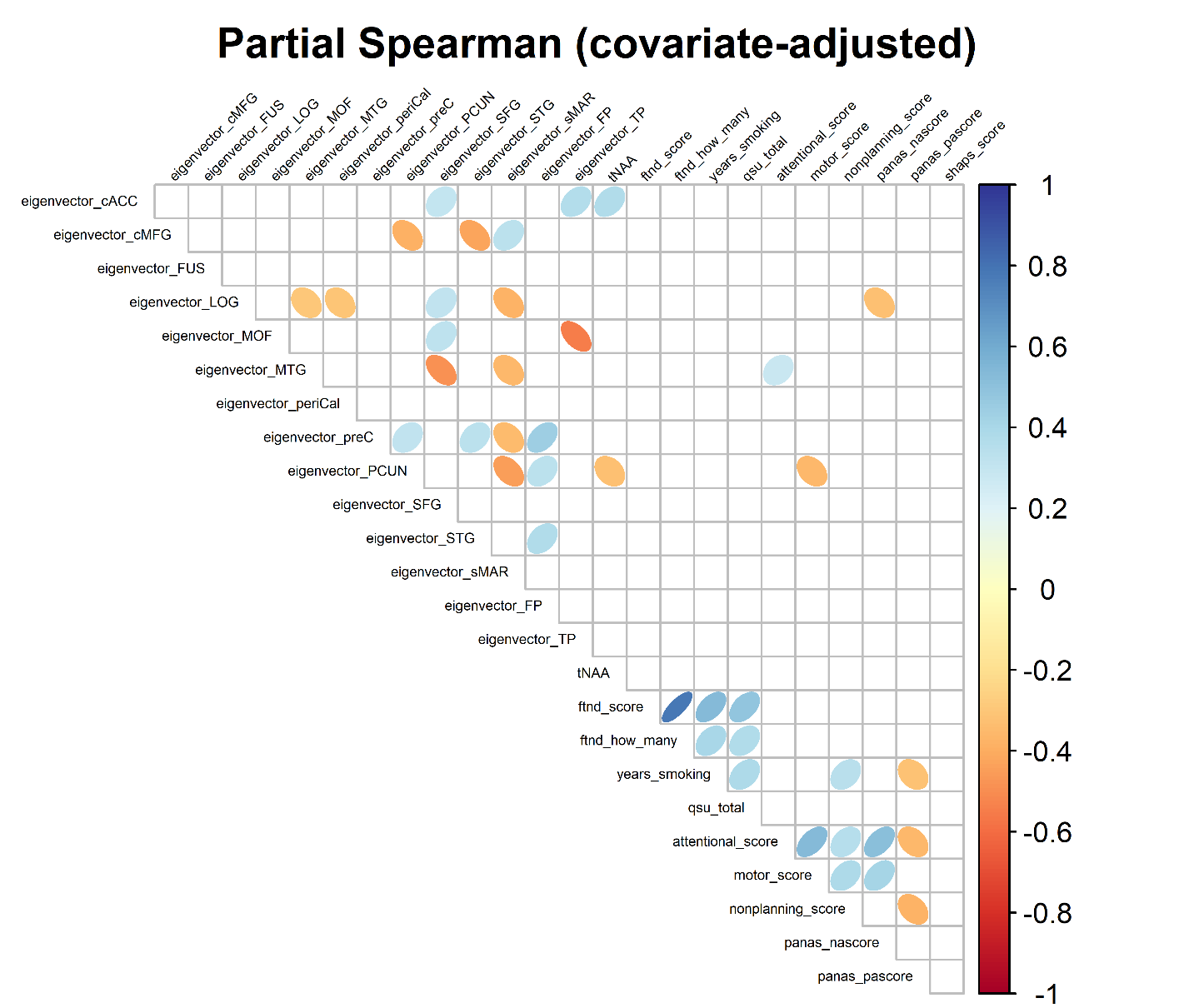


**Supplementary Figure 3**. Partial Spearman rank correlation matrix results between regional network metrics and smoking-related questionnaires. Blue colors indicate positive correlations while orange-red colors indicate negative correlations. Only significant correlations (not FDR corrected) are shown.

## *Breakdown of smoking status*

To provide some insight into the heterogeneity of the smoker group, here we have separated the smokers based on the range of cigarettes smoked per day as answered in the FTDN questionnaire.

**Supplementary Table 3**. Smokers grouped by range of cigarettes smoked per day along with the mean years of smoking and mean age.

|  | N | Mean Smoking Years  (M ± SD) | Mean Age [y]  (M ± SD) |
| --- | --- | --- | --- |
| 10 or less | 25 | 28 ± 15 | 48 ± 14 |
| 11 – 20 | 19 | 28 ± 11 | 45 ± 8 |
| 21 – 30 | 6 | 40 ± 11 | 53 ± 9 |
| 31 or more | 1 | 36 ± NA | 58 ± NA |

## *Group data for Glu, GSH, and GABA*

To provide some insight into the variance and variability of the measure Glu (PRESS), GSH and GABA (HERMES) at TE = 80 ms, boxplots are provided below. The same number of subjects as in the tNAA results presented in the manuscript are used in these comparisons: HC (n = 48), SM (n = 46).


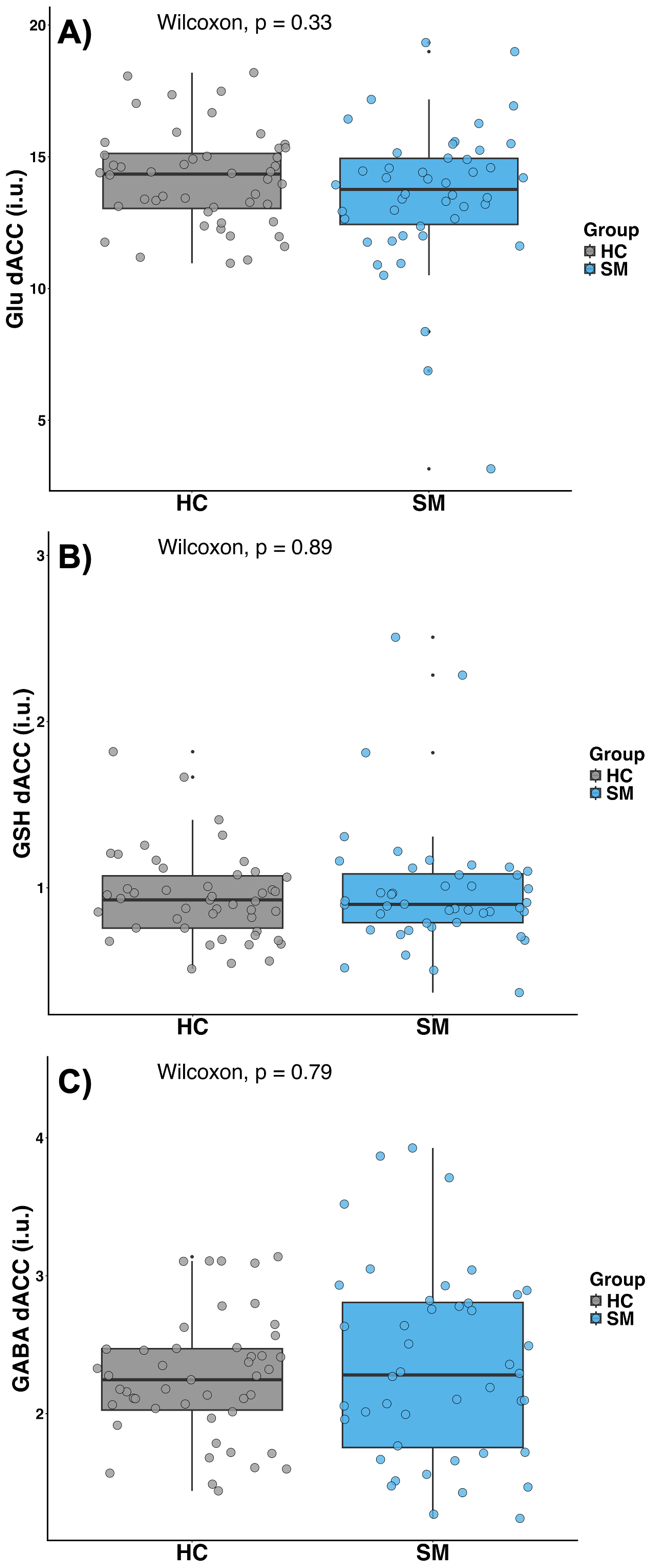


**Supplementary Figure 4.** Boxplots for A) Glu, B) GSH, and C) GABA in the dACC of the HC and SM groups. MRS measure values are water-scaled, CSF-corrected and reported as institutional units (i.u.). Uncorrected Wilcoxon p-value shown.
